# MiniMed 780G™ advanced hybrid closed-loop system performance in Egyptian patients with type 1 diabetes across different age groups: evidence from real-world users

**DOI:** 10.1186/s13098-023-01184-w

**Published:** 2023-10-17

**Authors:** Nancy Samir Elbarbary, Eman Abdel Rahman Ismail

**Affiliations:** 1 Department of Pediatrics, Faculty of medicine, Ain shams University, 25 Ahmed Fuad St. Saint Fatima, Cairo, 11361 Egypt; 2 Department of Clinical Pathology, Faculty of medicine, Ain shams University, Cairo, Egypt

**Keywords:** Type 1 Diabetes, Automated insulin delivery (AID), Advanced hybrid closed loop (AHCL), Minimed™ 780, Glucose management indicator, Time in range (TIR)

## Abstract

**Background:**

Advanced hybrid closed loop (AHCL) system provides both automated basal rate and correction boluses to keep glycemic values in a target range.

**Objectives:**

To evaluate the real-world performance of the MiniMed™ 780G system among different age groups of Egyptian patients with type 1diabetes.

**Methods:**

One-hundred seven AHCL system users aged from 3 to 71 years were enrolled. Data uploaded by patients were aggregated and analyzed. The mean glucose management indicator (GMI), percentage of time spent within glycemic ranges (TIR), time below range (TBR) and time above range (TAR) were determined.

**Results:**

Six months after initiating Auto Mode, patients spent a mean of 85.31 ± 22.04% of the time in Auto Mode (SmartGuard) and achieved a mean GMI of 6.95 ± 0.58% compared with 7.9 ± 2.1% before AHCL initiation (p < 0.001). TIR 70–180 mg/dL was increased post-AHCL initiation from 63.48 ± 10.14% to 81.54 ± 8.43% (p < 0.001) while TAR 180–250 mg/dL, TAR > 250 mg/dL, TBR < 70 mg/dL and TBR < 54 mg/dL were significantly decreased (p < 0.001). After initiating AHCL, TIR was greater in children and adults compared with adolescents (82.29 ± 7.22% and 83.86 ± 9.24% versus 78.4 ± 7.34%, respectively; p < 0.05). The total daily dose of insulin was increased in all age groups primarily due to increased system-initiated insulin delivery including auto correction boluses and basal insulin.

**Conclusions:**

MiniMed^™^ 780G system users across different age groups achieved international consensus-recommended glycemic control with no serious adverse effects even in challenging age group as children and adolescents.

**Supplementary Information:**

The online version contains supplementary material available at 10.1186/s13098-023-01184-w.

## Introduction

The lifelong goal of diabetes care is the early maintenance of glucose levels as close to normal as possible during the course of the disease and thus, delaying or possibly preventing devastating long-term diabetes complications [1].

The development of automated insulin delivery (AID) systems, have recently become an integral part of diabetes management. These advanced hybrid closed loop (AHCL) systems utilize an algorithm that automatically adjusts insulin delivery via an insulin pump based on real-time sensor glucose levels [2]. Currently, the MiniMed 780G system is the most advanced insulin pump system approved for the treatment of people with type 1 diabetes mellitus (T1DM) aged from 7 to 80 years. The system enables the personalization of glucose goals with an adjustable target setting as low as 100 mg/dL (5.5 mmol/L) [3]. The advanced SmartGuard algorithm in the MiniMed 780G system automates and personalizes the delivery of basal insulin by adjusting every five minutes, 24 h a day. This latest system also includes an advanced algorithm that automatically corrects highs every five minutes through autocorrection dosing, in addition to protecting against lows [4, 5].

Previous studies on 780G system showed a reduction in the variability of the outcomes achieved across different countries and across different age groups [4, 6]. Recent research has analyzed AHCL systems in kids and teens in different settings and indicated increased time in goal range [7–11]. The algorithm was also safe and performed well in adults in supervised settings and even for toddlers [12, 13].

Continuous glucose monitoring (CGM) systems have recently moved beyond mere blood glucose monitoring by providing both real-time and predictive glycemic data. CGM devices provide a broad spectrum of additional glucose management metrics, including proportions of time in range (TIR), time below range (TBR), time above range (TAR), and glucose variability (GV), that are at hand to person with diabetes and their health-care providers for individualizing the diabetes management and for making real-time treatment modifications [14].

Optimizing glycemic control for preschool children with T1DM and in the first months after onset is crucial for their future, both with respect to acute and long-time diabetes complications [15]. Furthermore, puberty-related physiological and hormonal changes that affect insulin action and insulin requirements, as well as a variety of behavioral factors is thought to contribute to under performances in youth with T1DM [16]. Cardiovascular disease (CVD) is the leading cause of morbidity and mortality in adults with T1DM, and increasing evidence demonstrates that CVD develops in childhood [17]. Moreover, meeting hemoglobin A1c (HbA1c) targets appears to be difficult, as only 10–15% of individuals diagnosed with T1DM before the age of 18 had an HbA1c within the target range in early adulthood [18]. For all these reasons, it is necessary to achieve and maintain stringent glycemic control in a safe way in all age groups living with diabetes.

Age-specific challenges to address in automated systems include the small insulin doses needed, often well below 10 U per day [19], the large difference in physiological insulin needs in different parts of the day, significant day-to-day variation in insulin needs and safety concerns to avoid accidental insulin dosing [20]. Including all age groups in clinical studies for diabetes technology development will ensure that these systems are better able to support the needs of all people living with T1DM. In addition to testing AHCL systems across a wide age spectrum, it is also important to ensure that these systems can be accessed by people with T1DM regardless of their prior therapy. Therefore, the aim of this real-world study was to evaluate the performance of the MiniMed™ 780G system among Egyptian patients with T1DM under free-living conditions as regards glycemic control and safety outcomes across different age groups. To the best of our knowledge, no previous studies assessed this system in African countries /MENA region among children, adolescents and adult patients with T1DM. So, the preliminary performance of the system in real-world settings was evaluated and analyzed.

## Materials and methods

This is a prospective, single-arm and open-label study where patients with T1DM were defined according to International Society for Pediatric and Adolescent Diabetes (ISPAD) guidelines [21]. Insulin Aspart (NovoRapid®, Novo Nordisk, Copenhagen, Denmark) was used in all patients on AHCL system. One-hundred seven Egyptian patients with T1DM aged from 3 to 71 years switching to AHCL system were enrolled and evaluated for MiniMed™ 780G system. Each patient or their legal guardians provided consent before participation for their data to be aggregated. Institutional review board (IRB) approval was obtained. Reporting of the study conforms to Consolidated Standards of Reporting Trials 2010 statement [22].

Data uploaded by MiniMed™ 780G system users to CareLink™ personal software over 6 months were analyzed to identify baseline, demographic and system use characteristics after initiating AHCL system. Two time periods were considered; the period before Auto-mode initiation of AHCL system was enabled for the first time (pre-AHCL) and the period after Auto-mode initiation of AHCL system was enabled for the first time (post-AHCL). Baseline sensor glucose (SG) data in this analysis refer to pre-AHCL SG outcomes. As described in several publications, only patients with ≥ 14 days of SG data were used to determine CGM-derived metrics [6, 23, 24]. All available data were included, irrespective of whether the system was in AHCL control or in open-loop (i.e. following an AHCL exit triggered by either the system or the user).

CGM derived glycemic metrics which includes the mean percentage of TIR between 70 and 180 mg/dL (3.9–10.0 mmol/L), TAR > 180 mg/dL (> 10.0 mmol/L) and > 250 mg/dL (> 13.9 mmol/L) as well as TBR 70 mg/dL (3.9 mmol/L) and 54 mg/dL (3.0 mmol/L) were determined for the overall 24-hour day. The mean SG levels, coefficient of variation (CoV) and GMI were also assessed, as well as the sensor use, percentage of time spent in AHCL, number of self-monitored blood glucose (SMBG) measurements, insulin delivery patterns, and the system settings (i.e., glucose target and active insulin time (AIT) and insulin consumed in users with 14 or more days of SG data before and after initial Auto mode start were determined. Baseline and follow-up visits were carried out; data were downloaded at each visit. System settings post-AHCL were identified in different age groups and their impact on TIR, TAR, TBR, post-AHCL was explored. All participants were asked to announce meals, calculate carbohydrate amounts and pre-bolus before meals.

The primary outcome was the mean percentage of time (TIR) with glucose level between 70 and 180 mg/dL (3.9–10.0 mmol/L) post-AHCL initiation. The secondary outcomes were the mean percentage of mean SG, GMI, CoV, TAR and TBR. Safety endpoints were the number of severe hypoglycemia or diabetic ketoacidosis (DKA).

### Statistical analysis

Analysis of data was done using Statistical Program for Social Science (IBM SPSS) version 27 (IBM Corporation, Armonk, NY, USA). Kolmogrov-Smirnov test was used to examine the normal distribution of variables. Quantitative variables were described in the form of mean and standard deviation. Qualitative variables were described as number and percent. Comparison of parametric quantitative variables between two groups was done using Student *t*-test. In order to compare quantitative parametric variables between the three age groups, one way Analysis of Variance (ANOVA) followed by post hoc analysis using Least significant difference (LSD) test were used. Qualitative variables were compared using Chi-square (X^**2**^) test or Fischer’s exact test when frequencies were below five. A p value < 0.05 was considered significant in all analyses.

## Results

### Baseline clinical characteristics of the studied population

This study included T1DM 780G™ AHCL system users (n = 107) living in Egypt who had ≥ 14 days of SG data both pre- and post-AHCL. Their ages ranged from 3 to 71 years (51 males and 56 females) with median (IQR) diabetes duration 5 (1.5–10) years; 79 (73.8%) patients used multiple daily injections and 28 (26.2%) patients used open loop insulin pump (sensor augmented pump-predictive low glucose management [SAP-PLGM]). The study population was further divided into three groups according to age; patients with age ranged from 11 to 18 years (n = 66) with median (IQR) 14.5 (12–16) years including 35 (53%) males, patients > 18 years (n = 20) with median (IQR) 30.5 (29–42) years including 16 (61.5%) males and patients < 11 years (n = 21) with a median (IQR) 7 (4–8.5) years including 11 (52.4%) males. The latter group included 8 patients < 7 years with a median age 4.8 years (range, 3–6 years).

### Impact of initiating AHCL on glycemic control (MiniMed™ 780G system usability performance) among all users

MiniMed™ 780G users were observed during the study period and the average set and reservoir change was 3.7 ± 1.4 days and 3.1 ± 0.6 days, respectively. During this time, mean sensor wear increased from 80.72 ± 29.17% to be 85.94 ± 19.0% post-AHCL initiation (p = 0.009). Patients spent a mean of 85.31 ± 22.04% of the time in Auto Mode (SmartGuard). The number of SMBG measurements decreased from 3.03 ± 1.6 to 2.85 ± 1.02 from pre- to post-AHCL initiation but non-significant (p = 0.052). There was 1.0 ± 0.8 AHCL exits per week, including 0.4 ± 0.3 triggered by the system and 0.5 ± 0.4 triggered by the users.

Six months after initiating Auto Mode, average SG improved significantly in all users from 183 ± 20.3 mg/dL to 151.7 ± 16.9 mg/dL (p < 0.001). Patients achieved a mean GMI of 6.95 ± 0.58% after AHCL initiation compared with 7.9 ± 2.1% before Auto-mode initiation (p < 0.001). The CoV% decreased from 58.9 to 34.1% post -AHCL (p < 0.001).

**Fig. 1:**
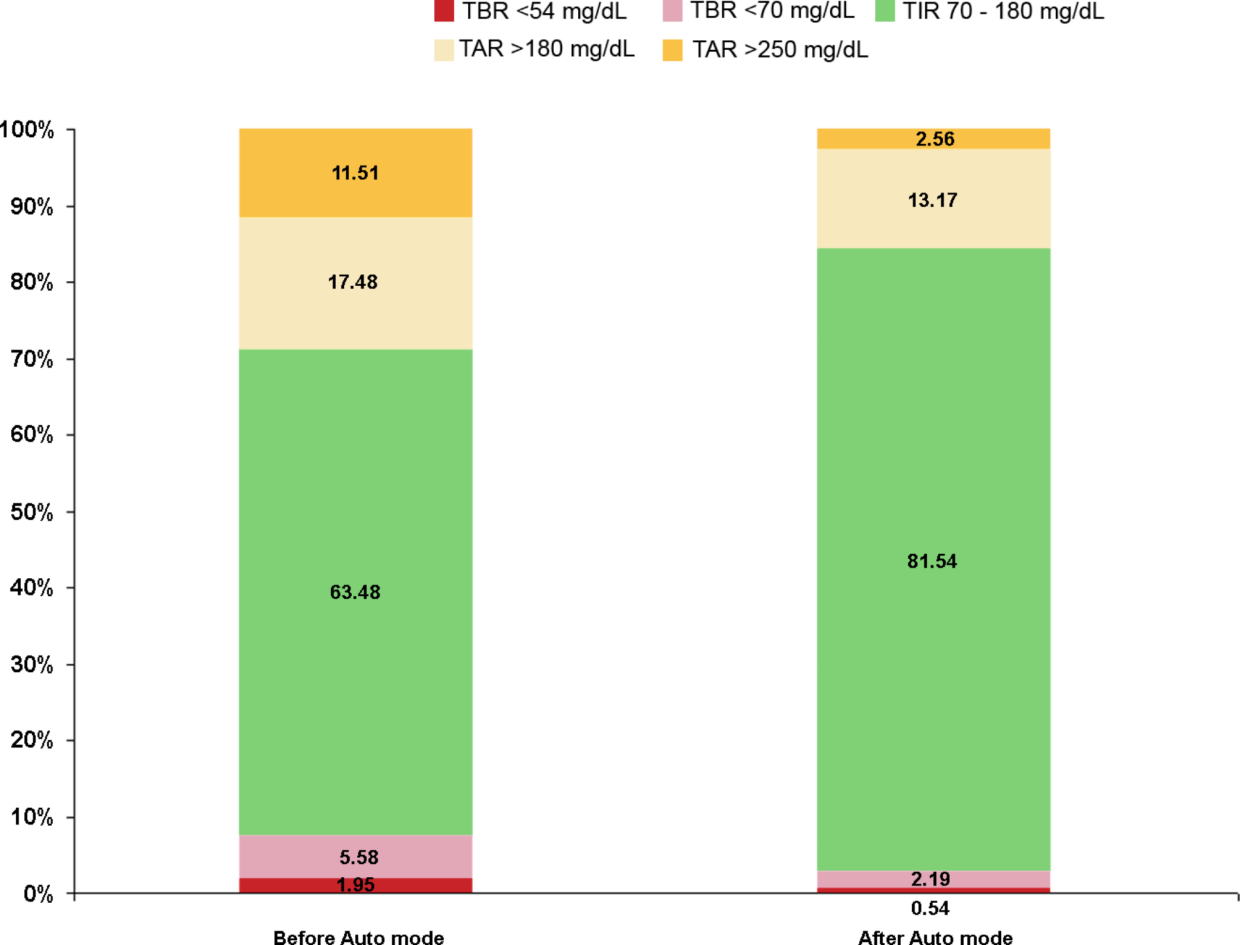
MiniMed™ 780G AHCL system performance showing glucose control before and after Auto-mode initiation among all patients with type 1 diabetes. Glucose values are shown as percentage spent in ranges. TBR: time below range; TIR: Time in range; TAR: Time above range

It was found that TIR 70–180 mg/dL was increased post-AHCL initiation from 63.48 ± 10.14% to 81.54 ± 8.43% (p < 0.001) with a mean TIR increment by 28.4%. The TAR 180–250 mg/dL was decreased from 17.48 ± 7.39% to 13.17 ± 6.15% and TAR > 250 mg/dL also decreased from 11.51 ± 10.80% to 2.56 ± 1.21% with a mean reduction of TAR 180–250 mg/dL by 24.5% and TAR > 250 mg/dL by 67.9% throughout the follow-up. The TBR < 70 mg/dL decreased from 5.58 ± 3.14% to 2.19 ± 1.11%% and TBR < 54 mg/dL decreased from 1.95 ± 1.68% to 0.54 ± 0.31% after initiating AHCL when compared with pre-AHCL initiation (p < 0.001 for all; Fig. 1).

The mean total daily dose (TDD) of insulin increased in all users from 32.41 ± 10.59 units/day pre-AHCL initiation to 35.17 ± 11.42 units/day post-AHCL (p = 0.002). This increase was mainly driven by auto corrections that accounted for 6.53 ± 3.13 units of insulin per day, representing 18.9% of TDD and 33.9% of total boluses together with system-initiated basal insulin delivery representing 45.3% of TDD. Both automated basal rate and correction boluses kept glycemic values in a target range.

### MiniMed™ 780G system performance among different age groups

As shown in Table 1, after initiating AHCL, all users achieved the glycemic treatment goals of GMI ≤ 7.0% (6.73 ± 0.40% for children and 6.71 ± 0.54% for adults versus 7.0 ± 0.58% in adolescents, p = 0.005). TIR 70–180 mg/dL was increased in all age groups post-AHCL compared with pre-AHCL. TIR 70–180 mg/dL was greater in children and adults compared with adolescents (82.29 ± 7.22% and 83.86 ± 9.24% versus 78.4 ± 7.34%, respectively; p = 0.010). The glucose target of 100 mg/dL (5.6 mmol/L) and an AIT of 2 h were set for both adolescents and adults while the glucose target 120 mg/dL (6.7 mmol/L) and AIT of 3 h were set for children during the study period. No difference in TIR among different age groups was observed between patients who were on multiple daily injections (MDI) versus open loop insulin pump before AHCL.

**Table 1:** MiniMed™ 780G system settings, usability, and glucometrics among different age groups of patients with T1DM after Auto-mode initiation of AHCL system

Variable	All users (n = 107)	< 11 years (n = 21)	11–18 years (n = 66)	> 18 years (n = 20)	Overall p between 3 age groups	p1	p2	p3
**BG calibration** (n/day)	2.85 ± 1.02	2.84 ± 0.48	2.95 ± 1.10	2.81 ± 1.13	0.865			
**Average SG** (mg/dL)	151.74 ± 16.87	146.00 ± 12.44	155.31 ± 17.74	145.14 ± 13.68	0.037	0.162	0.904	0.017
**GMI** (eA1C %) **GMI** (eA1C mmol/moL)	6.95 ± 0.58 52.6 ± 7.5	6.73 ± 0.40 50.4 ± 7.4	7.0 ± 0.58 55.3 ± 7.9	6.71 ± 0.54 50.1 ± 7.3	0.005 0.006	0.031 0.033	0.992 0.991	0.024 0.025
**CoV** (%)	34.1 ± 8.3	35.2 ± 8.7	38.9 ± 9.8	32.7 ± 8.1	0.023	0.256	0.667	0.028
**TIR 70–180 mg/dL** (%)	81.54 ± 8.43	82.29 ± 7.22	78.4 ± 7.34	83.86 ± 9.24	0.010	0.113	0.791	0.018
**TBR** < **70 mg/dL** (%)	2.19 ± 1.11	3.29 ± 1.50	1.88 ± 0.91	1.86 ± 0.98	< 0.001	< 0.001	< 0.001	0.995
**TBR < 54 mg/dL** (%)	0.54 ± 0.31	1.00 ± 0.69	0.29 ± 0.11	0.18 ± 0.09	< 0.001	< 0.001	< 0.001	0.366
**TAR 180–250 mg/dL** (%)	13.17 ± 6.15	12.71 ± 4.72	13.95 ± 6.12	11.6 ± 6.55	0.332			
**TAR > 250 mg/dL** (%)	2.56 ± 1.21	0.71 ± 0.50	5.48 ± 2.22	2.50 ± 1.32	0.075			
**Total daily dose** (U/day)	35.17 ± 11.42	12.24 ± 4.79	47.94 ± 10.72	45.35 ± 12.82	< 0.001	< 0.001	< 0.001	0.588
**Bolus amount** (U/day)	19.22 ± 8.76	8.23 ± 2.21	32.14 ± 9.46	24.94 ± 8.44	< 0.001	< 0.001	< 0.001	0.003
**Auto correction amount** (day)	6.53 ± 3.13	1.64 ± 0.32	7.07 ± 3.17	5.48 ± 2.86	< 0.001	< 0.001	< 0.001	0.070
**Auto Basal/Basal amount** (day)	15.95 ± 7.93	4.01 ± 1.95	15.80 ± 6.11	20.41 ± 8.98	< 0.001	< 0.001	< 0.001	0.012
**Smart Gaurd /week Auto Mode** (%)	85.31 ± 22.04	88.57 ± 23.46	82.71 ± 22.49	87.18 ± 22.17	0.507			
**Glucose target**, n (%)								
100 mg dL (5.6 mmol/ L) 110 mg dL (6.1 mmol/ L) 120 mg dL (6.7 mmol/ L)	70 (65.4) 23 (21.5) 14 (13.1)	0 (0) 7 (33.3) 14 (66.7)	53 (80.3) 13 (19.7) 0 (0)	17 (85.0) 3 (15.0) 0 (0)	< 0.001	< 0.001	< 0.001	0.894
**Sensor wear** (%)	85.94 ± 19.00	90.71 ± 24.34	82.50 ± 21.24	86.04 ± 17.90	0.298			
**Active insulin time**, n (%)								
2 h > 2 to 3 h > 3 to 4 h > 4 h	72 (67.3) 27 (25.23) 6 (5.61) 2 (1.86)	2 (9.5) 11 (52.4) 6 (28.6) 2 (9.5)	55 (83.3) 11 (16.7) 0 (0) 0 (0)	15 (75.0) 5 (25.0) 0 (0) 0 (0)	< 0.001	< 0.001	< 0.001	0.401
**Carbohydrates (**gr/day)	162 ± 33.2	132.6 ± 31.1	196.1 ± 39.3	150.4 ± 34.5	< 0.001	< 0.001	< 0.001	< 0.001
**ICR (**gr)	11.1 ± 2.9	16.6 ± 3.1	6.4 ± 2.1	7.3 ± 2.5	< 0.001	< 0.001	< 0.001	0.309
**Exit from AHCL per patient** (n/week)	1.0 ± 0.8	1 ± 0.5	2.0 ± 0.9	1 ± 0.6	< 0.001	< 0.001	0.715	< 0.001

T1DM: type 1 diabetes mellitus; AHCL: Advanced Hybrid Closed Loop System; BG: blood glucose; SG: sensor glucose; GMI: Glucose management indicator; eA1C: estimated A1C; CoV: coefficient of variation; TIR: time in range; TBR: time below range; TAR: time above range; ICR: insulin to carb ratio

P1: Comparison between T1DM patients < 11 years and 11–18 years

P2: Comparison between T1DM patients < 11 years and > 18 years

P3: Comparison between T1DM patients 11–18 years and > 18 years

### Dietary consumption (meals) or daily carbohydrate during study period

Meals and snacks were chosen by participants and were not restricted post-AHCL initiation. This was reflected in the average daily carbohydrates consumption estimates of 132.6 ± 31.1 gr/day, 196.1 ± 39.3 gr/day and 150.4 ± 34.5 gr/day in children, adolescents and adults, respectively; p < 0.001 (Table 1). Strengthening of insulin to carb ratio (ICR) was observed post- AHCL initiation reaching 11.1 ± 2.9 compared with 16.7 ± 3.7 pre-AHCL initiation (p < 0.001).

### Insulin delivery during AHCL

Insulin requirements varied widely among different age groups, with TDD in the cohort of children < 11 years as low as 12.24 ± 4.79 units/day compared with high TDD 47.94 ± 10.72 units/day and 45.35 ± 12.82 units/day in adolescents and adults, respectively (p < 0.001). For all age groups, the bolus amount per day, auto correction amount per day, auto basal/basal amount per day tended to increase during AHCL period (Table 1). This was true regardless of whether modality of insulin delivery before AHCL initiation.

### Safety outcomes

There were no serious adverse events among all the studied age groups, and the full AHCL period was completed for all participants with no episodes of severe hypoglycemia or DKA. Skin irritations related to sensor use occurred in five participants and resolved by local cream.

## Discussion

This study was designed to be broadly inclusive to the vast majority of T1DM population which represents unique challenges present at various life stages. Both prior published evidence and our findings suggest that some of the hurdles on the way to normoglycemia could be addressed by the new automated Medtronic system. Our results revealed that the use of MiniMed™ 780G AHCL system led to the improvement in TIR in all users and all age subgroups regardless of baseline HbA1c. This originated mostly from hyperglycemia and hypoglycemia reduction by the greatest percent likely due to automated basal insulin adjustments during the whole day and the hourly automatic correction boluses, which correct hyperglycemia during the day in apparent compensation for missed or incorrect meal boluses.

In this study, a significant reduction of the glucose variability and of HbA1c was noted. The use of this two-factor glycemic control assessment provides a more comprehensive picture of glycemia with CGM-derived metrics [4, 25–27]. Thus, the findings of this study demonstrated that Auto Mode allows a greater number of individuals with diabetes to achieve ADA and international consensus-recommended glycemic goal and all target percentages were on the average within the recommended ranges by literature consensus and no severe hypoglycemia or DKA episodes were recorded.

More specifically, we found that in the generally well-controlled adult group, the algorithm was able to significantly increase TIR and also reduce time in hypoglycemia < 70 mg/dL to below 2%. This percent time in hypoglycemia falls well below the recommendations for clinical targets recently set by an International Consensus Group [28] of 4%. This improvement was achieved while maintaining the mean glucose concentration at 145 mg/dL.

In comparison, children usually spend nearly half of the overnight hours during standard therapy with sensor glucose readings > 180 mg/dL. Parents of children are often fearful of hypoglycemia and may permit hyperglycemia to allay worries of dangerous low glucose levels, especially overnight. Parental fear may be precluding these children from achieving target glucose levels [29–31]. Previous research showed that HCL systems achieve the greatest TIR overnight when algorithms do not need to contend with food intake and physical activity [32].

Of note, we found that metabolic control was optimal (TIR > 78.4%), without increasing hypoglycemia, when AHCL settings were stricter (glucose target for SG level of 100 mg/dL (5.6 mmol/L) and an AIT of 2 h. Teenagers showed good technology adherence with optimal TIR maintained better over time.

AIT in our patients was set at 2 h with a target glucose level of 100 mg/dL for adolescents and adults and the autocorrect function activated. Using similar settings, Beato-Vibora et al. [33] used AHCL MiniMed™ 780G system and found these settings were not reflected by a significant increase in the proportion of time spent in hypoglycemia. The recommended target of 100 mg/dL is also proposed for the majority of users, while a less strict target of 110 mg/dl is recommended when age is < 15 years or when concerned about exacerbation of retinopathy if glucose levels are reduced rapidly and in case of hypoglycemia anxiety [34].

In the current study and in the study conducted by Beato-Vibora et al. [33], switching to the MiniMed™ 780G system was associated with an increase in the frequency of sensor use, possibly motivated by the AHCL system’s ability to respond to hyperglycemia when the CGM signal is available. Importantly, in both studies by Beato-Vibora et al. [33] and Seget et al. [35], sensor-augmented pump with low-glucose suspend (SAP-LGS) or predictive low-glucose suspend (SAP-PLGS) was used before switching to the MiniMed™ 780G system.

Report of real-world MiniMed 670G system use in Europe demonstrated substantial benefits for diabetes management regardless of baseline glycemic control (i.e., a GMI level of < 7.0% versus > 8.0%) [25]. This was also found in a more recent study by Lepore et al. [36] where switching to an AHCL MiniMed™ 780G system lead to a rapid improvement in glycemic control lasting for up to six months independently of previous insulin treatment and baseline conditions. In another study, AHCL initiation in adults with T1DM naive to CSII and CGM technologies significantly and safely improved their glycemic control. Time spent with glucose levels in target range increased from 69.3 ± 12.3% at baseline to 85.0 ± 6.3% at 3 months in the AHCL group while remained unchanged in the MDI + BGM group [37].

Within our cohort, the improvement in the glycemic control of our patients was associated with an increase in TDD from 32.41 ± 10.59 units/day pre-AHCL initiation to 35.17 ± 11.42 units/day post-AHCL (p = 0.002) in all users. This increase was mainly driven by auto corrections representing 18.5% of TDD and 33.9% of total boluses. Both automated basal rate and correction boluses kept glycemic values in a target range. This was comparable to other studies using the same AHCL system [26, 33, 38]. In a one year prospective observational study after starting AHCL, there was a slight increase in TDD per kg of body weight, but the percent of basal insulin was unchanged [39].

In line with our findings, Da Silva et al. [ 6 ] showed that the use of the 780G system in their patients was associated with a significant increase in TDD, which was probably associated with a lower degree of glycemic control at the baseline (mean glucose concentration 162.2 mg/dL, GMI 7.2% and 63.4% of time spent in the target range). This is in contrast to another study [35] where patients had better glycemic control at baseline and did not require an increase in TDD. This observation is worth emphasizing, given that the maintenance of TDD at the lowest possible level is important in preventing cardiovascular complications [17].

Petrovski et al. [38] reported that ICR modifications, automated bolus correction in addition to automated basal insulin delivery, as well as optimizing glucose target and AIT, effectively distributed the insulin delivery according to patients’ individual requirements, resulting in better glycemic outcome with minimal increase in TDD.

The real-world performance of the MiniMed™ 670G system in Europe [25] showed that users with baseline GMI of more than 8.0% had a significant increase in their TDD by 25.1% and their percentage of basal insulin delivered was increased by 19.1% after Auto Mode initiation. In contrast, basal insulin was reduced by 3.9% for the groups with a baseline GMI < 7.0%. Thus, real-world analyses [6, 25] and our study revealed a significant role for bolus insulin delivered, alongside automated basal insulin delivery to maintain baseline GMI < 7.0%.

During open-loop use (pre-AHCL), it is well-known in youth to lower basal rate settings (40.1% of TDD) compared with older individuals (48.3% of TDD) [40]. This practice has been attributed to the aim of lowering hypoglycemia risk in pediatric T1DM and compensating with more frequent meal and correction boluses. In the study of Arrieta et al. [40], following AHCL initiation, both youth and adults cohorts were closer to a 1:1 ratio, with system-initiated insulin delivery in the younger group mirroring that in adults (53.5% and 57.6%) driven by the algorithm with approximately the same amount of auto correction bolus as a percentage of all boluses (22.0% and 21.9%, respectively). These results are similar to those reported for the system in Collyns et al. [5] (25.1%) and Carlson et al. [4] (22.0%), yet substantially less than that observed in the FLAIR trial [26] (36%). However, direct comparison between these studies is limited because of differences in participants’ ages [40]. The amount of automated bolus per day highlights behavioral issues that prevent the best glycemic outcomes. These issues may include failure to bolus before meals or omitting meal boluses entirely. Ideally, the percentage of automated correction boluses should be in the low to-mid 20% range [40]. Recently, it has been shown that adolescents using the MiniMed™ 780G system with a preset of three personalized fixed carbohydrate amounts can reach international targets of glycemic control. Therefore, it may be a valuable alternative to precise carbohydrate counting in MiniMed™ 780G users who are challenged by it [41].

In our study, TIR 70–180 mg/dL was increased post-AHCL in all age subgroups and was greater in children and adults compared with adolescents. All users across different ages achieved the glycemic treatment goals of GMI < 7.08%. Our current results indicated improvement in glycemic control even in toddlers and preschoolers on MiniMed™ 780G AHCL system.

In screening variables for statistical associations with higher TIR, several demographic factors were noted as important; increase in age was associated with a 2.5% points increase in TIR for users aged > 55 years versus those aged ≤ 15 years, and male users had on average 0.9% higher TIR relative to female users. Although the changes were statistically significant, the overall outcomes across all ages and genders were well within the recommended guidelines [28] and the traditional gap in control in the younger versus older population [18, 42] is diminished with the AHCL system [40].

The differences in the age structures and models of insulin therapy of the analyzed patients are worth emphasizing, given that according to literature, achieving the ISPAD/ADA glycemic targets in the pediatric population could be much more challenging than in adults [43]. The use of AHCL in infants, toddlers and preschool children is still largely restricted to clinical trials. To date, only a few small studies have evaluated closed-loop systems in children younger than the age of 6 years [12, 44–46].

Notably, the evidence from clinical trials suggests that AHCL with AID can increase TIR, especially overnight, among very young children [44]. It has been reported that MiniMed™ 670G system use for 3 months by children 2–6 years of age versus open-loop therapy for 2 weeks was safe and helped to improve glycemic control, similar to use observed in older cohorts with T1DM [45]. Similarly, one randomized controlled crossover trial compared closed-loop with standard open-loop insulin pump therapy only from 10 pm to 12 pm on two consecutive days at an inpatient clinical research center. A trend toward a higher overnight TIR (70–200 mg/dL) in the closed-loop group has been shown, although this was not significant [46].

Recently, Pulkeinee et al. [12] has evaluated the safety and impact of MiniMed 780G™ system on glycemic outcome in 2 to 6 years old children with T1DM and showed that AHCL use was associated with improvements in glycemic control.

Another study reported a case of a 9 years old boy who started using MiniMed ^TM^ 780G with a TDD of 8.5 units that has dropped down to 5.7 units, with Auto Mode still running almost 100% of the time [47]. Furthermore, Tornese et al. [13] conducted a retrospective analysis of all children < 7 years of age with T1DM who were on the Medtronic MiniMed™ 780G system for at least 6 months with SmartGuard feature (Auto Mode). They reported that the use of this AHCL system is safe also with a TDD of insulin < 8 units and soon after diagnosis. The authors concluded that MiniMed™ 780G AHCL system should be considered a good therapeutic option for children age < 7 years from the onset of T1DM, also with a total insulin daily dose < 8 units, as with low body weights and in the remission phase. Our results further supported the above mentioned data and the idea that there should be no age limitations on who has access to this technology. Because they have previously met the suggested HbA1c targets or because their HbA1c levels are high, potential users should not be disqualified.

Strengths and at the same time possible limitations of this study are the broad age-range of the sample, going from toddlers and school-aged children to adults as well as the heterogeneity of previous therapeutic schemes. However, the real-life clinical practice setting is an important strength of our study. Another limitation of this study is the small number of enrolled patients, and the lack of a control arm and therefore, these findings need to be confirmed in larger multicenter studies with extended follow-up.

In conclusion, MiniMed™ 780G system users in real-world conditions across different age groups achieved international consensus-recommended glycemic control with no serious adverse effects. Children and adolescents with T1DM using the MiniMed™ 780G system achieved glycemic targets mirroring the achievements of the adult population using the system while maintaining safety from hypoglycemia or DKA. Thus, it is apparent that more stringent glycemic control is obtainable for a broad age range of individuals with T1DM, with time spent below range remaining within the recommended safe threshold. This provides a compelling case for increasing access to these systems to people with T1DM in all age groups. The findings from this analysis will potentially guide the optimal use of the MiniMed™ 780G system and facilitate meaningful improvements in safe glycemic control. Larger clinical trials of longer duration are required to expand on experience with this system for different age groups.

### Electronic supplementary material

Below is the link to the electronic supplementary material.

Supplementary Material 1

Supplementary Material 2

Supplementary Material 3

## Data Availability

The data that support the findings of this study are available from the corresponding author upon reasonable request.
