# Supplementary material for: MiniMed 780G™ advanced hybrid closed-loop system performance in Egyptian patients with type 1 diabetes across different age groups: evidence from real-world users
Source: Diabetol Metab Syndr. 2023 Oct 17;15:205. doi: 10.1186/s13098-023-01184-w (PMC10580510; doi:10.1186/s13098-023-01184-w)
Supplement: Supplementary file 1 — Supplementary Material 1 [file 13098_2023_1184_MOESM1_ESM.docx]

**Highlights**

- This is the first clinical trial assessing AHCL system in a broad age range of T1DM population.
- AHCL system improved mean glucose values, glycemic variability and time in ranges.
- MiniMed™ 780G allows all patients to reach the international targets for T1DM without compromising safety.
- Confidence in the system’s performance was demonstrated by the high use of the sensor
- More stringent glycemic control is obtainable for a broad age range of individuals with T1DM with no serious adverse effects
