# Supplementary material for: MiniMed 780G™ advanced hybrid closed-loop system performance in Egyptian patients with type 1 diabetes across different age groups: evidence from real-world users
Source: Diabetol Metab Syndr. 2023 Oct 17;15:205. doi: 10.1186/s13098-023-01184-w (PMC10580510; doi:10.1186/s13098-023-01184-w)
Supplement: Supplementary file 2 — Supplementary Material 2 [file 13098_2023_1184_MOESM2_ESM.docx]

**Title page**

**MiniMed 780G^TM^ Advanced Hybrid Closed-Loop System Performance in Egyptian Patients with Type 1 Diabetes across Different Age Groups: Evidence from Real-World Users**

Nancy Samir Elbarbary^1*^, Eman Abdel Rahman Ismail^2^

^1^Department of Pediatrics, Faculty of medicine, Ain shams University, Cairo, Egypt.

^2^Department of Clinical Pathology, Faculty of medicine, Ain shams University, Cairo, Egypt.

**Authors:**

****Nancy Samir Elbarbary***

Highest academic degree: MD Pediatrics.

Professor of Pediatrics, Pediatrics Department, Ain Shams University.

***Eman Abdel Rahman Ismail***

Highest academic degree: MD Clinical Pathology.

Consultant of Clinical Pathology, Clinical Pathology Department, Ain Shams University.

***Correspondence:**

Dr. Nancy Samir Elbarbary

25 Ahmed Fuad St. Saint Fatima, Heliopolis, Cairo 11361, Egypt

Fax: 0020224177712

Tel: 0020101117914

Email: [nancy_elbarbary@yahoo.com](mailto:nancy_elbarbary@yahoo.com)

Email: [nancy_elbarbary@med.asu.edu.eg](mailto:nancy_elbarbary@med.asu.edu.eg)

ORCID identifier: http://orcid.org/0000-0001-8713-0657

**Running title:** Effectiveness of AHCL system among different age groups

**Keywords:** Type 1 diabetes, Automated insulin delivery (AID), advanced hybrid closed loop (AHCL), Minimed™ 780, glucose management indicator, time in range (TIR).

**Tables:** 1

**Figures:** 1

**Abstract word count:** 248

**Text word count:** 4790

**Conflict of interest**: Nothing to declare

**Financial support**: None
