# Supplementary material for: MiniMed 780G™ advanced hybrid closed-loop system performance in Egyptian patients with type 1 diabetes across different age groups: evidence from real-world users
Source: Diabetol Metab Syndr. 2023 Oct 17;15:205. doi: 10.1186/s13098-023-01184-w (PMC10580510; doi:10.1186/s13098-023-01184-w)
Supplement: Supplementary file 3 — Supplementary Material 3 [file 13098_2023_1184_MOESM3_ESM.docx]

**Submission Letter**

Dear Editor,

The authors of the manuscript entitled “**MiniMed 780G^TM^ Advanced Hybrid Closed-Loop System Performance in Egyptian Patients with Type 1 Diabetes across Different Age Groups: Evidence from Real-World Users**” are honored to present their manuscript for publication and we hope you find it satisfactory. In recent years, the development of closed-loop systems, which link insulin delivery to sensor glucose levels, has started to transform management of type 1 diabetes mellitus (T1DM). These closed-loop systems utilize an algorithm that automatically adjusts insulin delivery via an insulin pump based on real-time sensor glucose levels. This glucose-responsive automated insulin delivery more closely replicates normal physiology. Including all age groups in clinical studies for diabetes technology development will ensure that these systems are better able to support the needs of all people living with T1DM. In addition to testing AHCL systems across a wide age spectrum, it is also important to ensure that these systems can be accessed by people with T1DM regardless of their prior therapy.

The aim of this real-world study was to evaluate the performance of the MiniMed ^TM^ 780G system among Egyptian patients with T1DM under free-living conditions as regards glycemic control and safety outcomes across different age groups. To the best of our knowledge, no previous study assessed this system in African countries /MENA region among children, adolescent, and adult patients with T1DM. So, the preliminary performance of the system in real-world settings was evaluated and analyzed.

The corresponding author, on behalf of all authors, hereby states that all authors have contributed to the manuscript in significant ways, have reviewed and agreed upon the manuscript content. The material submitted is novel and is not under editorial consideration in another journal.

Corresponding Author,

Dr. Nancy Samir Elbarbary

Professor of Pediatrics

Department of Pediatrics

Diabetes Unit, Ain Shams University, Faculty of Medicine.

Cairo 11361, Egypt

Email: [nancy_elbarbary@yahoo.com](mailto:nancy_elbarbary@yahoo.com)

ORCHID identifier: http://orcid.org/0000-0001-8713-0657

The authors would like to suggest the following reviewers:

1. Dr. Samia Ammar

[smammar@yahoo.com](mailto:smammar@yahoo.com)

2. Dr. Lamis Tawfik

[lamistawfik@gmail.com](mailto:lamistawfik@gmail.com)

3. Dr. Yasser Darwich

[yasserdarwich@gmail.com](mailto:yasserdarwich@gmail.com)

4. Dr. Rania El-Farrash

[raniafarrash@hotmail.com](mailto:raniafarrash@hotmail.com)

5. Dr. Ashraf Abou Elnasr

[elnasrashraf@hotmail.com](mailto:elnasrashraf@hotmail.com)
